# Nudging healthcare workers: assessing the impact of pre-booked appointments on influenza vaccination uptake

**DOI:** 10.3389/fpubh.2025.1701139

**Published:** 2025-11-21

**Authors:** Chiara Daicampi, Elisabetta Cesaro, Taisiia Kolmykova, Mario Degan, Vincenzo Baldo

**Affiliations:** 1 Department of Medicine, University of Padua, Padua, Italy; 2 Healthcare Profession Department, Padua University Hospital, Padua, Italy; 3 Department of Cardiac, Thoracic and Vascular Sciences, School of Medicine and Surgery, University of Padua, Padua, Italy

**Keywords:** influenza vaccination, vaccination uptake, healthcare workers, nudges, invitation letter

## Abstract

**Background:**

Influenza vaccination coverage among healthcare workers in Italy remains low compared with international benchmarks. Evidence on effective and scalable interventions in hospital settings is limited.

**Methods:**

We conducted a clustered quasi-experimental study in a large Italian university hospital, comparing the 2024/25 campaign to 2023/24. Hospital cost centers (*n* = 277) were non-randomly allocated to intervention (personalized letter with a pre-scheduled on-site appointment; 130 centers, 2,967 healthcare workers) or control (standard information; 147 centers, 1,577 healthcare workers). Administrative records provided uptake. We estimated Difference-in-Differences models at the cost-center level, weighting by center size and clustering standard errors at cost-center level, with subgroup analyzes by profession, gender, and age.

**Results:**

Overall coverage increased from 16.0% in 2023/24 to 25.2% in 2024/25. The DiD analysis indicated a significant effect of invitation letters (+4.0 percentage points). Stratification showed heterogeneous responses: the intervention was particularly effective among nurses, female workers, and mid-aged staff, while no effect was observed among physicians, the youngest, and the oldest age groups.

**Conclusion:**

Personalized invitation letters with pre-scheduled appointments represent a simple, scalable, and resource-efficient strategy to increase influenza vaccination uptake among HCWs. However, the effect was not homogeneous across subgroups, highlighting the importance of tailoring communication strategies to different professional and demographic profiles.

## 1 Introduction

Seasonal influenza is an acute respiratory infection caused by influenza viruses and remains a major public health concern worldwide (1). Influenza viruses evolve continuously, causing substantial morbidity and mortality each year, particularly among vulnerable populations such as the older adults, children, pregnant women, and individuals with chronic conditions (1). Vaccination remains the most effective preventive measure, yet coverage rates vary widely across countries and rarely achieve internationally recommended targets. In the EU/EEA, influenza vaccination coverage among older adults ranged from 5.6 to 78% during the 2021–22 season, with a median of 48.18%. Denmark reported the highest uptake, nearing the EU target of 78% (2). Vaccination among healthcare workers (HCWs) has been extensively studied due to their dual role in protecting themselves and their patients. However, uptake remains inconsistent. In the UK, only 36.5% of frontline HCWs were vaccinated between September and December 2024. Physicians generally reported higher adherence (58–79%), except in Croatia (32.6%), whereas nurses consistently showed lower rates (16–45%), with Finland as an outlier at 84.1% (3, 4). A recent meta-analysis (5) estimated average global coverage around 40%, confirming the challenge worldwide. Barriers to vaccination are multifactorial. Limited knowledge about immunization remains an important determinant: an Italian study reported that after a two-hour training session, 75% of nurses expressed a positive attitude toward vaccines, yet uptake remained modest, at 57% among coordinators and only 18% among nurses (6). Vaccine hesitancy also plays a role, with concerns about vaccine effectiveness, fears that vaccination might cause influenza (5, 7) perceptions of influenza as a mild illness (8), and appeals to personal autonomy or the maintenance of a “strong and healthy body” (9).

A broad range of strategies have been implemented to increase HCW vaccination uptake, typically classified into educational and promotional interventions, incentives, organizational strategies, policies, and combined approaches (10).

For example, a “free-flu-shot” campaign combining free vaccination, advertising, and incentives (e.g., prizes and gifts) achieved coverage rates of 80% in 2018 (11). Another program incorporating lectures, email reminders, and recruitment by key opinion leaders raised coverage to 52.8% compared to 26.5% in controls (12). In Italy, an operational protocol that included on-site vaccination services resulted in a 17% increase in targeted units, compared to a 1.5% increase in control units (13). Multifaceted approaches—such as promotional campaigns, extended vaccination hours, and small incentives (e.g., an additional day off)—have consistently demonstrated effectiveness in different healthcare settings (14, 15).

Evidence on the use of personalized invitations with pre-booked appointments in large hospitals is limited, and little is known about their heterogeneous effects. This study evaluates a personalized invitation letter with a pre-assigned vaccination appointment, implemented in a large university hospital. Its originality lies in combining fixed appointments with a Difference-in-Differences design to provide a causal estimate of effectiveness, while also assessing differential effects across age groups, gender, and professional categories.

## 2 Materials and methods

### 2.1 Study design and setting

We conducted a clustered quasi-experimental study to evaluate the effectiveness of personalized invitation letters with pre-booked vaccination appointments on influenza vaccination coverage among HCWs in a large university hospital in north-eastern Italy (1,682 beds; ~7,000 employees). The study compared the 2024/25 campaign with 2023/24 at the hospital cost-center level. Allocation to intervention or control was non-random and followed a predefined operational logic established by the hospital management board. Cost centers were assigned to the intervention when on-site clinic capacity and timetabling allowed delivery of pre-booked appointments near the unit, and when infection-risk profiles prioritized early access for patient-facing wards. Remaining eligible centers were assigned to the control arm. Allocation decisions were finalized before extraction of 2024/25 uptake data and were not informed by prior vaccination rates or interim results. The analysis focused on physicians, nurses, and healthcare assistants, the three largest professional groups within the hospital workforce.

### 2.2 Intervention

In total, 277 cost centers were included. In October 2024, staff in the intervention group (130 cost centers, 2,967 employees) received a personalized invitation letter, addressed by name and containing a pre-assigned vaccination appointment with date, time, and the clinic closest to the staff member’s unit. The letter specified that appointments could be rescheduled by email and included a detachable refusal slip to report reasons for declining vaccination.

The control group (147 cost centers, 1,577 employees) received only general information on the vaccination campaign, including available locations and opening hours. Vaccination data were collected between October 2024 and February 2025. A copy of the invitation letter is reported in Supplementary material. The above allocation criteria reflected feasibility and infection-control priorities rather than expected vaccination behavior, and no baseline uptake information was consulted at the time of assignment.

### 2.3 Data collection and analytic sample

Administrative records provided aggregated vaccination uptake for the 2023/24 and 2024/25 campaigns. We constructed balanced panel cells at the level cost center × year × subgroup, where subgroups were defined by professional role (physician, nurse, healthcare assistant), gender, or age group (≤35, 36–55, ≥56). Starting from all observed cells, we applied pre-specified cleaning rules:

- Implausible shares (>1) were set to missing;
- Subgroup cells not observed in both years were excluded to ensure a balanced two-period panel.

This procedure yielded 850 analytic cells used in the regression analysis.

Additionally, data on refusals to participate in the campaign and the reasons behind this choice were collected for the group who received the invitation.

### 2.4 Outcome measure

The primary outcome was vaccination coverage in each analytic cell, defined as the proportion of HCWs vaccinated in the respective cost center, year, and subgroup.

### 2.5 Statistical analysis

We first produced descriptive statistics for the overall sample and by professional role, year, and intervention status.

We then estimated cross-sectional regression models for the 2023 and 2024 campaigns, both with and without covariates. To account for persistence in vaccination behavior, additional 2024 models were estimated including 2023 vaccination rates as a covariate.

We also employed a Difference-in-Differences (DiD) approach comparing vaccination uptake between invited and non-invited cost centers before (2023/24 campaign) and after (2024/25 campaign) the intervention. Formally, the model can be expressed as:


Yit=α+βInvitedi+γPostt+δ(Invitedi×Postt)+εit.

Where Y*it* is the vaccination coverage in cost center *i* at year *t*, Invited*i* indicates whether the cost center received the invitation letters, Post*t* is a dummy for the 2024 campaign, and the interaction term (Invited*i* × Post*t*) captures the net causal effect of the intervention (*δ*).

All models were weighted by cost-center size, and standard errors were clustered at the cost-center level; robustness was confirmed when clustering at the department level. As a sensitivity check, we estimated an augmented DiD model including baseline vaccination coverage in 2023 to account for persistence in behavior. With only two periods, the parallel-trends assumption could not be formally tested; however, its plausibility is supported by (i) the absence of baseline differences between groups in 2023 and (ii) the stability of results in baseline-adjusted models.

To assess effect heterogeneity, we estimated stratified DiD models and formal interaction models including triple terms (Invited × Post × Subgroup) by professional role (physician, nurse, healthcare assistant), age group (≤35, 36–55, ≥56), and gender. Corresponding p-for-interaction values are reported.

## 3 Results

A total of 3,070 invitation letters were distributed, of which 103 could not be delivered because the recipients were on long-term sick leave, maternity leave, or pregnancy, leaving 2,967 eligible employees. Among these, 993 returned a refusal letter for vaccination.

For the empirical analysis, data were aggregated by hospital cost center, professional role (nurses, physicians, healthcare assistants), and year. This produced 906 cost center–role–year combinations. We excluded 29 observations with inconsistencies in vaccination shares, 27 not observed in both years, and cost centers that could not be reliably matched across years. The final analytic sample consisted of 850 cost center–role–year observations, corresponding to 277 distinct cost centers.

Table 1 summarizes weighted characteristics of HCWs by treatment status. Invited units were slightly younger on average—particularly among nurses—whereas gender composition and professional mix were broadly similar across groups. These modest differences supported the use of adjusted regression models and of an analytical strategy comparing changes over time rather than relying solely on post-intervention differences. This approach ensures that estimated effects capture the impact of the invitation letters while accounting for any pre-existing imbalance and time-invariant unobserved factors.

**Table 1:** Descriptive statistics of HCWs by treatment status in the 2024/25 influenza campaign.

	Invited units (intervention group)Mean (SD)	non-invited units (control group)Mean (SD)
All		
Vaccinated (%)	24.7 (21.8)	18.5 (20.5)
Female (%)	75.8 (20.8)	77.2 (21.7)
Age group ≤35 (%)	31.6 (21.6)	16.4 (21.3)
Age group 36–55 (%)	49.7 (18.3)	53.6 (32.5)
Age group ≥56 (%)	18.7 (14.3)	30.0 (22.9)
Cost center size	14.3 (11.6)	7.23 (7.68)
Nurses		
Vaccinated (%)	19.6 (14.1)	15.9 (15.3)
Female (%)	81.1 (16.1)	82.7 (17.3)
Age group ≤35(%)	41 (21.3)	17.6 (22.6)
Age group 36–55(%)	44.7 (17.8)	53.1 (19.3)
Age group ≥56(%)	14.3 (10.6)	29.3 (20.3)
Cost center size	21.6 (13.1)	8.9 (8.9)
Physician		
Vaccinated (%)	35.9 (27.7)	29.8 (29.9)
Female (%)	55.2 (21.0)	51.4 (21.3)
Age group ≤35(%)	25.4 (16.1)	27.0 (22.2)
Age group 36–55(%)	54.0 (17.7)	52.0 (24.4)
Age group ≥56(%)	20.6 (15.3)	21.0 (22.2)
Cost center size	10 (7.8)	5.8 (6.8)
Healthcare assistant		
Vaccinated (%)	10.1 (13.8)	8.5 (15.7)
Female (%)	79.4 (20.8)	76.6 (23.1)
Age group ≤35(%)	14.5 (11.8)	6.7 (10.2)
Age group 36–55(%)	57.9 (16.7)	56.2 (25.7)
Age group ≥56(%)	27.6 (16.6)	37.1 (28.0)
Cost center size	9.8 (8.2)	5.1 (4.4)

Means weighted by cost-center size.

Nurses represented the largest subgroup, while physicians accounted for roughly one-fifth of the sample and healthcare assistants for the remainder. Invited staff were younger on average than those in control units, particularly among nurses.

In 2023, before the intervention, vaccination coverage was low and comparable across invited and non-invited cost centers (14.6% vs. 12.4%), with no statistically significant difference between groups. Between 2023 and 2024, uptake increased overall, but the rise was significantly larger among invited staff. In non-invited centers, coverage grew from 12.4 to 18.5% (+6.1 percentage point - pp, *p* = 0.002), while in invited centers it increased from 14.6 to 24.7% (+10.1 pp, *p* < 0.001). Accordingly, by 2024 mean uptake was 24.7% in the treatment group versus 18.5% in controls, with regression analysis confirming a significant advantage for invited centers (+6.2 pp, *p* = 0.005).

To assess whether the invitation letters drove this increase, we conducted cross-sectional analyzes restricted to the 2024 campaign. Supplementary Table S1 reports the unadjusted mean-comparison tests (first four columns) which already suggest a positive effect of the invitation, and multivariate models controlling for gender and age (last four columns), which confirm the result.

To verify that these findings are not driven by pre-existing differences between groups, Supplementary Table S2 presents analogous regressions for the 2023 baseline, when no invitation letters were sent.

Results show no significant differences between invited and non-invited centers, either in unadjusted or adjusted models. This supports the interpretation that the higher uptake observed in 2024 is attributable to the invitation letters rather than pre-existing imbalances.

We also adjust for the 2023 vaccination rate by cost center (Supplementary Table S3). These analyses, together with alternative model specifications and stratified estimates, serve as sensitivity checks confirming the robustness of our main findings. Taken together, these consistency checks indicate that the observed differences are not an artifact of model specification or sample composition, but a stable feature across analytical approaches. This baseline adjustment effectively absorbs any time-invariant unobserved factors that could otherwise bias the estimates. In other words, if systematic differences existed between invited and non-invited centers, they should have already emerged in 2023, when no intervention was in place. The absence of such disparities reinforces the credibility that the higher uptake observed in 2024 genuinely reflects the effect of the invitation letters rather than pre-existing imbalances.

Building on these findings, and to more formally isolate the causal effect of the intervention, we implemented a Difference-in-Differences (DiD) approach, which compares within-center changes in vaccination coverage between the two campaigns (Table 2).

**Table 2:** Difference-in-Differences estimates of the invitation effect on vaccination uptake, overall and by professional role.

Professional group	Did estimate	95%CI	*p*-value
Nurses	+4.5	1.0; 7.1	0.011
Physicians	0	−10.0; 10.1	0.995
Healthcare assistants	+3.4	−0.5; 7.4	0.09
Overall	+4.0	0.9; 7.1	0.012

Between 2023 and 2024, the DiD interaction term shows that invitation letters produced a statistically significant increase. When stratifying by professional profile, the intervention was particularly effective among nurses, the largest professional group, while no effect was detected among physicians. For healthcare assistants, the estimated effect was positive but did not reach conventional significance levels, possibly due to smaller sample size and lower baseline uptake.

Subsequently, we stratified the DiD by age group and by gender (Table 3).

**Table 3:** Difference-in-Differences estimates by age group and by gender.

Age group	Did estimate	95%CI	*p*-value
Age group ≤35	+0.6	−5.3; 6.5	0.83
Age group 36–55	+7.7	3.5; 11.6	0.000
Age group ≥56	+0.6	−4.9; 6.1	0.82
Gender			
Male	+3.4	−0.6; 7.4	0.1
Female	+5.0	0.1; 9.9	0.045

When stratifying the analysis by age group, the effect of the invitation letters was concentrated among mid-aged HCWs, while no meaningful changes were detected among the youngest and oldest groups. A similar pattern emerged by gender, with female workers showing a clearer response to the intervention compared with males. Overall, these additional analyzes indicate that the impact of invitation letters was not homogeneous across subgroups, but mainly driven by women and mid-aged staff.

### 3.1 Refusal of vaccination

HCWs who received the invitation letter but did not intend to get vaccinated had the option to return the letter, allowing for the collection of data on refusals to participate in the campaign and the reasons behind their decision. A total of 993 refusal letters were received. Among these, 11% expressed doubts about the vaccine’s efficacy and safety, while 14% cited concerns about potential adverse events. A further 4.8% reported other types of doubts and concerns, and 29% stated that they had already been vaccinated elsewhere. For the purpose of the analysis, these HCWs were classified as “non-vaccinated,” as it was not possible to verify vaccination uptake outside the hospital setting, which may imply a conservative estimate of the intervention effect.

Overall, 41% cited “other” reasons for declining vaccination. Among these, 78% provided no further details, 11% explicitly stated a lack of interest in vaccination, while a small percentage cited medical contraindications (2.7%) or reported having experienced adverse effects following previous vaccinations (3.7%).

## 4 Discussion

The influenza vaccination rate among HCWs in the University Hospital involved in this study was 16% during the 2023/24 season, comparable to the national average in Italy (11–16%) and to historical national rates from 2004 to 2007 (9–13%) (16). While consistent with past data, this rate remains markedly lower than the global average of 41.7%, with the highest coverage observed in the Americas (67.1%) (5). Unlike the United States, where influenza vaccination is mandatory in most hospitals (17), in Europe it remains a recommendation rather than a requirement. At the regional level, influenza vaccination rates were 20.2% in Italy during the 2022/23 season and 18.9% in 2023/24, while in the region where the hospital is located, they were slightly lower at 18.7 and 17.6%, respectively (18). Against this background, our findings document a positive trend, with an overall increase to 25.2% in the 2024/25 season.

Our DiD estimates suggest that personalized invitation letters with pre-booked vaccination appointments were associated with an increase in vaccination uptake among HCWs. While the parallel trend assumption underlying DiD cannot be formally tested due to the availability of only two periods, its plausibility is supported by the absence of baseline differences in 2023 and by the robustness of results when adjusting for baseline vaccination rates.

The estimated +4 percentage points from the DiD analysis may appear modest in absolute terms. However, when comparing final coverage rates in 2024/25, invited centers achieved 24.7% versus 18.5% in non-invited centers. This corresponds to a relative increase of about 33%, meaning that roughly one-third more HCWs were vaccinated thanks to the intervention. Evidence from previous systematic reviews confirms that reminder and invitation strategies are effective in increasing vaccination uptake among HCWs (5, 7), although precise effect sizes are rarely quantified. In the general population, a recent meta-analysis found that reminder and recall interventions increased coverage by 38.5% (95% CI: 28.9–48.9%) (19), suggesting that our findings are consistent with the broader evidence base.

The intervention showed differential effects by professional category. Physicians, who typically exhibit higher baseline vaccination rates and greater awareness of vaccine efficacy (20), consistent with findings by Barbara et al. (15), may have experienced a ceiling effect. In this group, alternative professional information channels and existing high propensity for vaccination may have reduced the marginal impact of the intervention. Nurses, in contrast, exhibited the strongest response, suggesting that structured reminders and facilitated access (e.g., pre-scheduled appointments, nearby clinics) are particularly effective for this group. Among healthcare assistants, the estimated effect was modest and not statistically significant, likely reflecting both smaller numbers and lower baseline knowledge about immunization, in line with evidence linking lower educational attainment to lower vaccine uptake (21, 22). Additional subgroup analyzes further revealed heterogeneity by gender and age. The effect was concentrated among women and mid-aged HCWs (36–55 years), while no relevant changes were observed among men, younger, or older workers. This pattern is consistent with previous evidence showing that female tend to display greater acceptance of preventive interventions (23), and that mid-aged workers often face both professional and family responsibilities, which may heighten their perceived benefits of influenza protection (24).

From a behavioral perspective, the intervention leveraged the principles of “nudging,” particularly the default effect, by assigning HCWs a fixed vaccination appointment. This reduced decisional inertia and made adherence the easiest option. Previous studies have shown that pre-booked appointments can improve vaccine uptake in the general population (24) but evidence in complex hospital settings has been limited.

Our results contribute new insights by demonstrating the feasibility of this approach in a large, heterogeneous healthcare workforce. This study was conducted in a single large tertiary hospital encompassing a wide range of specialties and HCWs profiles. This setting provides a diverse and representative sample within tertiary care.

In line with evidence from multifaceted interventions, organizational facilitation—such as on-site clinics and extended hours—remains crucial (10, 13–15).

From a policy perspective, our results suggest that interventions based on pre-booked appointments are simple, resource-efficient, and highly scalable. They may be particularly effective when targeted at professional groups and demographic subgroups where responsiveness is greatest—namely nurses, women, and mid-aged HCWs. Combining default appointments with on-site vaccination clinics could represent a pragmatic strategy for healthcare systems seeking to improve uptake while facing budgetary and workforce constraints.

This study has several limitations. Allocation of cost centers was not randomized but decided administratively, which precludes strict causal inference and leaves open the possibility of residual confounding. Moreover, we relied on aggregated administrative data rather than individual-level records, which restricted our ability to adjust for additional covariates such as previous vaccination history or contract type. The study was also conducted in a single hospital, limiting generalizability to other settings with different organizational cultures or baseline coverage rates. In addition, our Difference-in-Differences design spanned only two influenza seasons, preventing a formal test of the parallel trends assumption. Finally, because the intervention combined two elements—personalized letters and pre-scheduled appointments—we cannot disentangle their separate contributions to the observed uptake. Future research could isolate these elements to identify the most effective behavioral lever.

## 5 Conclusion

This study suggests that personalized invitation letters with pre-scheduled appointments may offer a feasible and low-cost approach to modestly increase influenza vaccination coverage among HCWs. By combining behavioral nudges with organizational facilitation, such interventions can contribute to incremental improvements in uptake, particularly among nurses and mid-aged staff.

However, given the persistently low overall coverage observed, these gains remain insufficient to ensure broad protection, highlighting the need for more comprehensive, multi-component strategies to strengthen influenza prevention among HCWs and patients. From a practical perspective, such low-cost, easily implementable measures could nonetheless be valuable components of broader institutional vaccination programs, supporting incremental progress in settings with limited resources or low baseline coverage.

## Data Availability

The data analyzed in this study is subject to the following licenses/restrictions: The anonymized dataset and replication code used for the analyzes are available from the corresponding author upon reasonable request. Requests to access these datasets should be directed to elisabetta.cesaro@studenti.unipd.it.
