# Supplementary material for: Nudging healthcare workers: assessing the impact of pre-booked appointments on influenza vaccination uptake
Source: Front Public Health. 2025 Nov 21;13:1701139. doi: 10.3389/fpubh.2025.1701139 (PMC12678282; doi:10.3389/fpubh.2025.1701139)
Supplement: Supplementary file 1 [file Supplementary_file_1.docx]

**Nudging healthcare workers: assessing the impact of pre-booked appointments on influenza vaccination uptake**

**Supplementary materials**

**C**O**NTENTS**

[**Supplemental Table 1** Cross-sectional regression estimates for the 2024/25 campaign](https://docs.google.com/document/d/1jIT6kJlrYvAy1ndzWN4paFUVivBFxQrM/edit#heading=h.gjdgxs)

[**Supplemental Table 2.** Vaccination uptake in 2023](https://docs.google.com/document/d/1jIT6kJlrYvAy1ndzWN4paFUVivBFxQrM/edit#heading=h.3znysh7)

[**Supplemental Table 3.** 2024/25 vaccination uptake models adjusted for 2023/24 baseline coverage, overall and by professional role.](https://docs.google.com/document/d/1jIT6kJlrYvAy1ndzWN4paFUVivBFxQrM/edit#heading=h.2et92p0)

**Supplemental Invitation Letter.** The invitation letter sent to HCWs with a pre-booked appointment.

| Independent variables | All | Nurses | Physicians | Healthcare Assistant | All | Nurses | Physicians | Healthcare Assistant |
| --- | --- | --- | --- | --- | --- | --- | --- | --- |
| Invited | 0.044**  (0.018) | 0.037*  (0.022) | 0.062  (0.069) | 0.051**  (0.024) | 0.056***  (0.018) | 0.057**  (0.025) | 0.061  (0.067) | 0.057**  (0.024) |
| Female |  |  |  |  | 0.009  (0.043) | -0.034  (0.064) | 0.059  (0.131) | 0.037  (0.048) |
| Age Group ≤35 |  |  |  |  | -0.010  (0.051) | -0.025  (0.062) | 0.068  (0.178) | 0.035  (0.129) |
| Age Group ≥ 56 |  |  |  |  | 0.095*  (0.053) | 0.101  (0.081) | 0.101  (0.081) | 0.099  (0.063) |
| Physicians | 0.324***  (0.035) |  |  |  | 0.324***  (0.039) |  |  |  |
| Healthcare Assistant | -0.053***  (0.018) |  |  |  | -0.066***  (0.020) |  |  |  |
| Constant | 0.155***  (0.013) | 0.159***  (0.014) | 0.466***  (0.055) | 0.096***  (0.017) | 0.124***  (0.046) | 0.162**  (0.063) | 0.400***  (0.131) | 0.029  (0.045) |
| Observations | 425 | 194 | 91 | 140 | 425 | 194 | 91 | 140 |
| R-Squared | 0.375 | 0.015 | 0.011 | 0.027 | 0.381 | 0.032 | 0.016 | 0.048 |

**Supplementary Table 1. Cross-sectional regression estimates for the 2024/25 campaign.** *** p<0.01, ** p<0.05, * p<0.1, Robust standard errors in parentheses *Notes: Estimates from univariate regressions (columns 1–4) and multivariable regressions controlling for gender and age (columns 5–8). Weighted by centre size.*

| Independent variables | All | Nurses | Physicians | Healthcare Assistant |
| --- | --- | --- | --- | --- |
| Invited | 0.019  (0.018) | 0.006  (0.022) | 0.048  (0.067) | 0.029  (0.026) |
| Female | 0.109***  (0.040) | 0.050  (0.040) | 0.294**  (0.040) | 0.053  (0.040) |
| Age Group ≤35 | -0.016  (0.048) | -0.035  (0.056) | 0.126  (0.192) | -0.081  (0.107 |
| Age Group ≥ 56 | 0.063  (0.048) | 0.030  (0.069) | 0.151  (0.148) | 0.051  (0.059) |
| Physicians | 0.271***  (0.040) |  |  |  |
| Healthcare Assistant | -0.013  (0.019) |  |  |  |
| Constant | -0.011  (0.044) | 0.059  (0.058) | 0.089  (0.094) | 0.024  (0.050) |
| Observations | 425 | 194 | 91 | 140 |
| R-Squared | 0.241 | 0.012 | 0.071 | 0.022 |

**Supplementary Table 2. Vaccination uptake in 2023** *** p<0.01, ** p<0.05, * p<0.1, Robust standard errors in parentheses *Notes: Multivariable regressions controlling for gender and age, restricted to the 2023 campaign. Weighted by centre size.*

| Independent variables | All | Nurses | Physicians | Healthcare Assistant | All | Nurses | Physicians | Healthcare Assistant |
| --- | --- | --- | --- | --- | --- | --- | --- | --- |
| Invited | 0.041***  (0.013) | 0.047***  (0.016) | 0.029  (0.049) | 0.037*  (0.019) | 0.044***  (0.015) | 0.055***  (0.018) | 0.032  (0.051) | 0.035*  (0.020) |
| %Vax. In 2023 | 0.673***  (0.046) | 0.763*** (0.069) | 0.629***  (0.075) | 0.619***  (0.080) | 0.677***  (0.046) | 0.760***  (0.069) | 0.646***  (0.075) | 0.616***  (0.080) |
| Female |  |  |  |  | -0.055*  (0.032) | -0.062  (0.044) | -0.107  (0.100) | -0.002  (0.043) |
| Age Group ≤35 |  |  |  |  | 0.002  (0.040) | 0.003  (0.043 | -0.001  (0.135) | 0.076  (0.113 |
| Age Group ≥ 56 |  |  |  |  | 0.031  (0.042) | 0.062  (0.056) | -0.051  (0.150) | 0.044  (0.052) |
| Physicians | 0.156***  (0.028) |  |  |  | 0.139***  (0.030) |  |  |  |
| Healthcare Assistant | -0.052***  (0.013) |  |  |  | -0.057***  (0.015) |  |  |  |
| Constant | 0.089***  (0.011) | 0.077***  (0.013) | 0.269***  (0.048) | 0.045***  (0.014) | 0.125***  (0.033) | 0.109**  (0.043) | 0.330***  (0.102) | 0.026  (0.040) |
| Observations | 425 | 194 | 91 | 140 | 425 | 194 | 91 | 140 |
| R-Squared | 0.660 | 0.482 | 0.481 | 0.404 | 0.662 | 0.490 | 0.489 | 0.409 |

**Supplementary Table 3. 2024/25 vaccination uptake models adjusted for 2023/24 baseline coverage, overall and by professional role.***** p<0.01, ** p<0.05, * p<0.1, Robust standard errors in parentheses *Notes: Regression models for 2024 vaccination uptake including the 2023 baseline coverage at cost-centre level as covariate. Weighted by centre size*

**Invitation letter**

Gentile collega,

Il Piano Nazionale di Prevenzione 2023-25 promosso dal Ministero della Salute, in linea con le raccomandazioni dell’Organizzazione Mondiale della Sanità, ha come obiettivo strategico il raggiungimento di una significativa COPERTURA VACCINALE ANTINFLUENZALE STAGIONALE per gli operatori delle strutture sanitarie ed assistenziali.

L’adesione volontaria alla campagna vaccinale rappresenta un elemento chiave a tutela della salute degli operatori e dei pazienti.

La Direzione dell’Azienda Ospedale-Università offre la possibilità di effettuare in orario di servizio la vaccinazione antinfluenzale presso le proprie strutture.

Per questo motivo, è invitato a presentarsi nella sede e data indicata:

| *Day of the year* | *Hour* | *Which ambulatory* |
| --- | --- | --- |

È pregato di presentarsi all’ambulatorio con l’allegata Scheda di registrazione consenso già precompilata e con il badge personale di riconoscimento.

Se fosse impossibilitato a partecipare nella data indicata, si chiede di inviare una e-mail all’indirizzo *professionisanitarie.aopd@aopd.veneto.it*; verrà ricontattato per programmare un nuovo appuntamento.

Qualora invece non desideri partecipare alla campagna vaccinale si chiede cortesemente di restituire per posta interna il presente invito alla Segreteria UOC Direzione delle Professioni Sanitarie, specificandone i motivi:

- dubbi su efficacia e/o sicurezza del vaccino
- timori di effetti collaterali/eventi avversi
- intenzione di effettuare la vaccinazione in altre sedi (es. medico di famiglia)
- altro: __________________________________________________________________

I dati raccolti serviranno a gestire le sedute, rivalutando gli assenti e i presenti ed a programmare al meglio la proposta vaccinale.

Distinti saluti.

Il Direttore Sanitario
